# Feature tracking and aging

**DOI:** 10.3389/fpsyg.2013.00427

**Published:** 2013-07-15

**Authors:** Rémy Allard, Sarah Lagacé-Nadon, Jocelyn Faubert

**Affiliations:** Visual Psychophysics and Perception Laboratory, School of Optometry, Université de MontréalMontréal, QC, Canada

**Keywords:** aging, motion, feature tracking, fractal rotation, second-order motion

## Abstract

There are conflicting results regarding the effect of aging on second-order motion processing (i.e., motion defined by attributes other than luminance, such as contrast). Two studies (Habak and Faubert, 2000; Tang and Zhou, 2009) found that second-order motion processing was more vulnerable to aging than first-order motion processing. Conversely, Billino et al. (2011) recently found that aging affected first- and second-order motion processing by similar proportions. These three studies used contrast-defined motion as a second-order stimulus, but there can be at least two potential issues when using such a stimulus to evaluate age-related sensitivity losses. First, it has been shown that the motion system processing contrast-defined motion varies depending on the stimulus parameters. Thus, although all these three studies assumed that their contrast-defined motion was processed by a low-level second-order motion system, this was not necessarily the case. The second potential issue is that contrast-defined motion consists in a contrast modulation of a texture rich in high spatial frequencies and aging mainly affects contrast sensitivity at high spatial frequencies. Consequently, some age-related sensitivity loss to second-order motion could be due to a lower sensitivity to the texture rather than to motion processing *per se*. To avoid these two potential issues, we used a second-order motion stimulus void of high spatial frequencies and which has been shown to be processed by a high-level feature tracking motion system, namely fractal rotation (Lagacé-Nadon et al., 2009). We found an age-related deficit on second-order motion processing at all temporal frequencies including the ones for which no age-related effect on first-order motion processing was observed. We conclude that aging affects the ability to track features. Previous age-related results on second-order and global motion processing are discussed in light of these findings.

## Introduction

Healthy aging induces several physiological, perceptual and cognitive changes. At the level of the visual system, several visual functions are found to decrease with advancing age, such as contrast sensitivity (Owsley et al., 1983), visual acuity (Weale, 1975; Owsley et al., 1983) and perceptual processing (Faubert, 2002). Visual perception is classically looked at in terms of low or high level perceptual functions. These would require different level of cognitive processing and hence, could be altered differentially by healthy aging of the visual system. Interestingly, Faubert (2002) suggested that the underlying physiological processes involved in both low and higher level perceptual functions are probably altered with aging, but that these age-related deficits should be more functionally apparent when processing higher level information. This explanation has been referred to as the “processing complexity hypothesis of aging” (Faubert, 2002). The rationale here is that when there are diffuse subtle neurobiological changes as a result of aging, some perceptual functions may still be performed at similar levels by the elderly because of the recruitment of alternate neural networks (e.g., McIntosh et al., 1999; Della-Maggiore et al., 2000; Bennett et al., 2001). However, when processing implicates larger neural machinery or requires larger simultaneous networks, performance breaks down. On this basis it is expected that higher-order processing will be more affected as it was shown for symmetry perception, inter-attribute spatial frequency discrimination and other functions.

From the complexity hypothesis perspective, it is interesting to study the effect of aging on two similar perceptual tasks that differ in processing complexity such as first- and second-order motion processing. First-order motion stimuli are defined by local variations of luminosity (Anstis and Mather, 1985; Chubb and Sperling, 1988, 1989; Cavanagh and Mather, 1989; Wilson et al., 1992), which can be processed directly by the well-known first-order motion system, that is, low-level energy-based spatiotemporal filtering. In contrast, second-order stimuli are those defined by other properties than luminance, such as contrast, polarity and orientation, presumably making them “invisible” to the first-order motion system. To perceive second-order motion, a given property (e.g., contrast) of a texture (i.e., the “carrier”) must first be locally estimated before its modulation can be globally integrated over space and time. This integration can either be performed by a low-level energy-based spatiotemporal filter (i.e., a second-order motion system) or by an actively tracking position shift of the texture (i.e., a feature tracking motion system). The second-order motion system would be low-level and analogous to the first-order motion system (i.e., motion extraction by energy-based spatiotemporal filtering). However, as opposed to first-order motion system, it would first require an extra processing step consisting in rectifying the texture modulation introducing energy at the spatiotemporal frequency of the texture modulation (Wilson et al., 1992; Lu and Sperling, 1995, 2001). Conversely, the feature tracking motion system would be high-level and would consist of a different processing strategy: identify the position of the texture modulation and attentively track the position shift over time (Cavanagh, 1992).

Three studies have evaluated the effect of aging on first- and second-order motion processing and found diverging results. Two of those (Habak and Faubert, 2000; Tang and Zhou, 2009) found that second-order motion processing was more vulnerable to aging than first-order motion processing, which is consistent with Faubert's complexity hypothesis. Conversely, Billino et al. (2011) found that aging affected first- and second-order motion processing by similar proportions, which suggests “more direct associations between functional decline and differential ageing of critical brain areas” (p. 3160). These three studies used contrast-defined motion as second-order stimuli. There are at least two potential issues when using such a stimulus to evaluate age-related sensitivity losses. The first issue pertains to the fact that the motion system processing contrast-defined motion varies depending on the stimulus parameters, such as interstimulus interval (Smith, 1994), texture contrast (Ukkonen and Derrington, 2000), modulation contrast (Seiffert and Cavanagh, 1999) and temporal frequency (Holliday and Anderson, 1994; Seiffert and Cavanagh, 1999; Allard and Faubert, 2008a). The three studies assumed that their contrast-defined motion was processed by a low-level second-order motion system. However, this was not necessarily the case. Furthermore, besides the fact that the motion system processing contrast-defined motion depends on many parameters, even the existence of a second-order motion system remains controversial. Some (Ukkonen and Derrington, 2000; Allard and Faubert, 2008a) have argued that contrast-defined motion can either be processed by the low-level first-order motion system due to non-linearities (which could explain the same age-related effect for first- and second-order motion processing found by Billino et al., 2011) or by a high-level feature tracking motion system (which could explain the specific age-related effect to second-order motion processing observed by Habak and Faubert, 2000 and Tang and Zhou, 2009), which questions the existence of a second-order motion system. The second potential issue is that contrast-defined motion requires the modulation of a texture rich in high spatial frequencies, such as noise or a high spatial frequency sine wave grating, and aging mainly affects contrast sensitivity to high spatial frequencies (Kline et al., 1983; Owsley et al., 1983; Morrison and McGrath, 1985; Crassini et al., 1988) Consequently, motion perception that requires the processing of high spatial frequencies could artificially induce an age-related sensitivity loss to second-order motion attributable to a lower sensitivity to the texture (i.e., carrier), rather than to the motion processing *per se*. As such, Billino et al. (2011) suggested that part of the previously reported age-related sensitivity loss specific to the second-order motion processing could be explained by age-related changes to the optics of the eye, which changes mainly affect contrast sensitivity at high spatial frequencies. However, high spatial frequencies are essential for the processing of contrast-defined motion.

**Figure 1:**
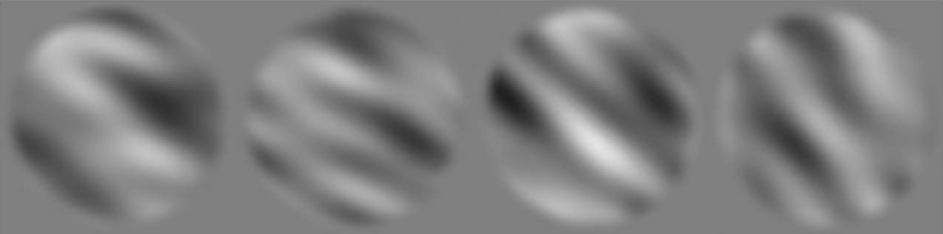
**Fractal rotation stimulus example**. Stimulus is rotating clockwise. From this 4-frames sequence, it can be seen that a noise frame is resampled at every frame. An example movie of these stimuli can be viewed in Lagacé-Nadon et al. (2009; Movie 2).

To avoid these two potential issues, the current study used a second-order motion stimulus proven to be processed by a high-level feature tracking motion system (Lagacé-Nadon et al., 2009), namely fractal rotation. This stimulus was originally introduced by Benton et al. (2007). We modified the stimulus (Lagacé-Nadon et al., 2009) to eliminate all high spatial frequency components from its composition. It is composed of successive noise frames rich in orientation cues changing over time resulting in a rotating percept (Figure 1). Since the noise is resampled at every frame (i.e., dynamic noise), there is no local luminance correlation between frames resulting in no net local luminance translation cue. As a result, fractal rotation is “invisible” to the first-order motion system, which is sensitive to luminance translation cues. Note that contrast-defined motion (the second-order stimuli used in the previous aging studies cited above), as fractal rotation, does not contain any net local luminance translation cues and should therefore be “invisible” to the first-order motion system. However, early non-linearities within the stimulus or the visual system can easily introduce luminance variations (Smith and Ledgeway, 1997), making such a second-order stimulus “visible” to the first-order motion system. As opposed to most second-order stimuli, fractal rotation does not consist in the modulation of a texture, so that even strong early non-linearities would not introduce any net luminance translation cues. This robustness to early non-linearities guaranties that fractal rotation cannot be processed by the first-order motion system. Indeed, to perceive rotation from fractal stimuli, one needs to track changes in orientation over time, rather than local luminance translations. As a result, fractal rotation is processed by the high-order feature tracking motion system (Lagacé-Nadon et al., 2009). We therefore chose fractal rotation as our second-order motion stimulus because (1) it is robustly “invisible” to the first-order motion system and, (2) it can be composed exclusively of low spatial frequencies, which are not or little affected by aging (Owsley et al., 1983), thereby minimizing visibility difference between younger and older adults.

Feature tracking requires observers to attentively track changes of a given property (e.g., position or orientation) and is therefore attention-based. Another attention-based motion task widely studied is multiple-object tracking (MOT). Performance to MOT tasks has been found to decline with age (Trick et al., 2005; Sekuler et al., 2008; Kennedy et al., 2009), which could reflect a decline of attention-based processing. However, it is difficult to determine precisely which process is affected by aging since the performance to a MOT task depends on many factors, such as working memory, the observer's strategy (e.g., eye gaze) and the ability to divide attention and maintain it over a long period of time. Conversely, feature tracking has the advantage of probing attention without directly soliciting all these factors. Thus, feature tracking appears to be relatively simple for an attention-based task and if there is a general age-related decline of attention, then this simple attention-based task should also be affected.

## Experiment 1: discrimination of direction of first-order and fractal rotation

Contrast sensitivities to motion direction discrimination of first-order and fractal rotation (Benton et al., 2007) were measured for two age groups. The use of a band-pass spatial filter to keep only spatial frequencies ranging between 0.125 and 0.5 cycles per degree (cpd) ensured that the observed decline in contrast sensitivity to motion direction is attributable to the effect of age on motion processing *per se*, rather than to diminished visibility of the noise pattern.

Contrast sensitivity was measured over a large range of temporal frequencies to reflect the band-pass and the low-pass sensitivity function of first-order and feature tracking motion processing. Measuring sensitivity over a large range of temporal frequencies is particularly relevant because aging affects motion sensitivity differently at different temporal frequencies. As such, Habak and Faubert (2000) reported a significant age-related decrease in motion sensitivity of first-order stimuli presented at low (2 Hz) and high (8 Hz) temporal frequencies, but not at a medium temporal frequency (4 Hz). These results underline the importance of looking at motion perception over a large range of temporal frequencies.

### Materials and methods

#### Subjects

Participants have been divided into two groups, the younger and the older adults groups. Ten individuals aged between 18 and 32 years of age (mean age 23.8 ± 5.01 years) and 12 individuals between 65 and 75 years old (mean age 68.46 ± 2.65 years) participated in the study. All participants needed to have a best corrected monocular visual acuity of at least 6/6. Subjects were required to have a good ocular health to be included and any subject with strabismus, amblyopia, cataract, age-related macular degeneration, glaucoma, cerebral vascular accident history or visual field dysfunctions was excluded. Subjects from the older adult group all had a complete visual examination done by an optometrist at the School of Optometry of Université de Montréal within the year before the experiment. Our protocol was approved by the university's research ethics board. Informed consent was given by each participant upon evaluation.

A Mini-Mental State Examination (MMSE) was administered to older participants prior to psychophysical evaluation. Average score on the MMSE for the older adult population was 29.5/30 ± 0.1946 (range: 28–30/30). All subjects were located in the 75th percentile or above for their age and educational level, except for two subjects who were located between the 50 and 75th percentiles (Crum et al., 1993). Ametropias, astigmatism and presbyopia were all corrected, after which measures of monocular and binocular acuity was performed to ensure optimal correction was obtained for the testing distance.

#### Apparatus and stimuli

Stimuli were generated by a Pentium 4 computer. Images were presented on a ViewSonic E90FB.25CRT computer screen using a Matrox Parhelia 512 graphic card. The Noisy-Bit method (Allard and Faubert, 2008b) implemented with the error of the green color gun inversely correlated with the error of the two other color guns made the 8-bit display equivalent to an analog display having a continuous luminance resolution. Mean luminance of the screen was 47 cd/m^2^ and refresh rate was 60 Hz. Each pixel possessed 1/32 degrees of visual angle at the viewing distance of 57 cm. The monitor was the only source of light in the room. A Minolta CS100 photometer interfaced with a homemade program calibrated the output intensity of each gun. Presentation time was 1 s to ensure optimal temporal integration for the older adult group (Raghuram et al., 2005; Bennett et al., 2007).

**Figure 2:**
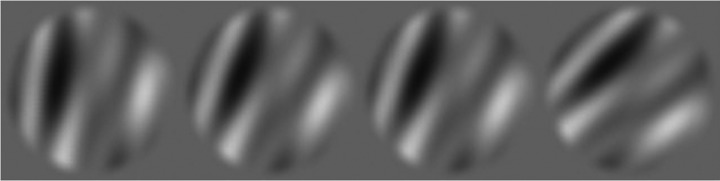
**First-order rotation stimulus**. Stimulus is rotating clockwise. From this 4-frames sequence, it can be seen that a single noise frame is rotated in time. An example movie of these stimuli can be viewed in Lagacé-Nadon et al. (2009; Movie 1).

In the present study, two types of stimuli were presented: first-order and fractal rotation (Benton et al., 2007; Lagacé-Nadon et al., 2009). Stimuli were composed of 1/f noise that was filtered as a function of the spatial frequency and orientation. The filtering in the spatial frequency dimension was done to minimize the visibility difference of the stimulus between young and old observers. Given the well-known age-related contrast sensitivity losses to high spatial frequencies (Owsley et al., 1983; Morrison and McGrath, 1985; Crassini et al., 1988; Tulunay-Keesey et al., 1988; Elliott et al., 1990), each noise frame was band-pass filtered to keep only low spatial frequencies ranging from 0.125 to 0.5 cpd. A 10 degrees wide orientation filter was applied to each presented noise frame, such that the image spatial structure was rich in orientation cues. A rotating stimulus was composed of successive noise frames that were rotated after being filtered in the spatial and orientation dimensions. The rotating direction (clockwise or counterclockwise) and initial orientation were randomized on each trial. The only difference between the first-order and fractal rotations stimuli was that a different noise sample was used at each frame (refreshed at 60 Hz) for the fractal stimulus and the same noise sample was used for the first-order stimulus. Thus, the first-order stimulus corresponded to a rotating noise image (Figure 2) and the fractal rotation corresponded to dynamic noise in which the orientation varies over time (Figure 1). Hence, for the first-order stimulus, the local motion direction could be determined by local luminance translation cues, which can be detected by the low-level first-order motion system. But for the fractal stimulus, the direction of local luminance translation cues was random due to the noise resampling at every frame. So to perceive fractal rotation, one needs to track changes in spatial structure (i.e., orientation) over time, rather than local luminance translations, so fractal rotation is processed by the high-order feature tracking motion system (Lagacé-Nadon et al., 2009). Both stimuli were presented within a circular aperture, subtending 8 degrees of visual angle and were displayed on a gray background.

#### Procedure

Before each session, subjects were adapted to room luminance testing condition for 20 min (Jackson et al., 1999). A single-interval, two-alternative forced-choice procedure was used in combination with a direction discrimination task (clockwise or counterclockwise). A 2-down-1-up staircase protocol was used to measure contrast thresholds (Levitt, 1971). Each staircase consisted of 10 reversals and thresholds corresponded to the geometric mean of the last 6 reversals. Each trial started with the apparition of the fixation bull's-eye to which participants were asked to maintain fixation. A feedback sound was provided to participants. Temporal frequencies of presented stimuli were 0.25, 0.5, 1, 2 circle rotations (360 degrees) per second (or r/s) for fractal rotation and 0.25, 0.5, 1, 2, 4, 6, 8 r/s for first-order rotation. Speeds of presented stimuli were determined based on the temporal frequency functions of both first-order and fractal rotation stimuli, as established in a previous study (Lagacé-Nadon et al., 2009). Each participant completed fifteen blocks of trials. The first four blocks consisted of practice trials to familiarize subjects with first-order and fractal rotation stimuli. The presentation order of the 11 block conditions (4 fractal and 7 first-order rotation speeds) was randomized. Trials and practice trials were divided into two equal testing sessions, which were conducted on separate days. Stimuli were viewed binocularly.

### Results and discussion

**Figure 3:**
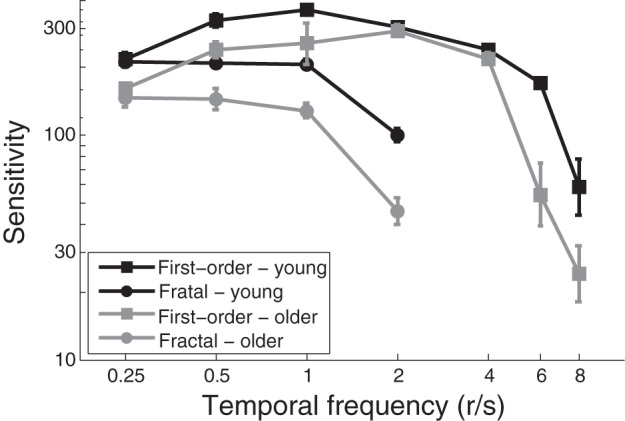
**Mean contrast sensitivity to first-order and fractal rotation as a function of temporal frequency**. Error bars represent the standard error of the mean.

Contrast thresholds for discrimination of direction of first-order and fractal rotation stimuli were obtained for each participant as a function of temporal frequencies. Group results for both younger and older adults are presented in Figure 3. Results are expressed in terms of contrast sensitivity, which was defined as the reciprocal of the contrast threshold. Consistent with our previous findings (Lagacé-Nadon et al., 2009), contrast sensitivity functions of first-order and fractal rotations were band-pass and low-pass in nature, respectively, which is consistent with our interpretation that first-order and fractal rotation stimuli are analyzed by the first-order and feature tracking motion systems, respectively.

As can be seen in Figure 3, different age-related sensitivity losses were observed with the two stimuli. Age-related sensitivity losses for the first-order rotation were not uniform, with sensitivity losses observed only at low (i.e., 0.25, 0.5, 1 r/s) and very high temporal frequencies (6, 8 r/s). As such, contrast sensitivity thresholds for first-order rotation at medium temporal frequencies (2, 4 r/s) were comparable between the younger and older adult group. These results are consistent with those obtained by Habak and Faubert (2000) who found an age-related sensitivity loss to first-order motion processing at low (2 Hz) and high (8 Hz) temporal frequencies, but not at a medium temporal frequency (4 Hz). Conversely, contrast sensitivity to fractal rotation was reduced at all temporal frequencies for the older adult as compared to the younger adult group. Importantly, an age-related sensitivity loss to fractal rotation was observed at a temporal frequency (2 r/s) at which first-order motion processing was unaffected. This shows that aging affects the feature tracking motion processing *per se*, and that the results cannot simply be explained by another general factor not directly related to the task.

These findings were statistically confirmed by performing a three-way repeated measure ANOVA (age × stimulus type × temporal frequency) on logarithmic contrast sensitivity values with stimulus type (2 levels: first-order and fractal rotation) and temporal frequency (4 levels: 0.25, 0.5, 1, and 2 r/s) as within-subject factors and age as between-subject factors (2 levels: younger and older adults). Analysis revealed a significant three-way interaction [*F*_(2.043, 40.852)_ = 5.399, *p* = 0.002]. Further two-way ANOVA were performed to examine interactions between age and stimulus type for each temporal frequency. At 0.25, 0.50, and 1 r/s, there was no interaction [*F*_(1, 20)_ = 0.390, *p* = 0.539 for 0.25 r/s, *F*_(1, 20)_ = 0.295, *p* = 0.593 for 0.50 r/s and *F*_(1, 20)_ = 0.292, *p* = 0.595 for 1 r/s], but a significant main effect of age was found [*F*_(1, 20)_ = 16.839, *p* = 0.001 for 0.25 r/s, *F*_(1, 20)_ = 11.258, *p* = 0.003 for 0.50 r/s and *F*_(1, 20)_ = 9.600, *p* = 0.006]. Consequently, it is not possible to determine whether the age-related sensitivity loss to fractal rotation was specific to fractal rotation motion processing at these temporal frequencies. At 2 r/s, however, a significant age x stimulus type interaction was identified [*F*_(1, 20)_ = 21.897, *p* < 0.001]. Independent samples *t*-test revealed significant effect of age for fractal rotation [*t*_(20)_ = 4.915, *p* < 0.001] but not for first-order rotation [*t*_(20)_ = 0.543, *p* = 0.593]. This shows an age-related impairment specific to fractal rotation motion processing at this medium frequency.

To evaluate age-related impairment to first-order rotation at higher temporal frequencies, another two-way repeated measures ANOVA has been performed with the temporal frequency as the within-subjects factors (3 levels: 4, 6, and 8 r/s) and age as the between-subjects variable (2 levels: younger and older adults). A significant interaction between age and speed of presented stimuli was found [*F*_(2, 40)_ = 3.313, *p* = 0.047]. Independent sample t-test revealed significant effect of age at 6 and 8 r/s for first-order motion [*t*_(20)_ = 3.588, *p* = 0.004 for 6 r/s and *t*_(20)_ = 2.149, *p* = 0.044 for 8 r/s) such that thresholds for discrimination of direction were higher for the older individuals. However, no significant effect of age was observed for first-order motion presented at 4 r/s [*t*_(20)_ = 0.996, *p* = 0.331].

To evaluate if there were common factors affecting the sensitivity to both first-order and fractal rotation motion processing, we evaluated the correlation between these thresholds for both age groups. The Spearman's correlation coefficients with variables independently ranked for each temporal frequency were 0.41 and 0.46 for young and older adults, respectively. These moderate correlations between first-order and fractal rotation thresholds can be explained by factors that affect both thresholds but vary between subjects. These factors could be low-level (e.g., contrast gain due to light scattering) or high-level (e.g., motivation or fatigue).

In sum, the main findings indicate significant effect of age on direction discrimination thresholds of fractal rotation at all temporal frequencies, and first-order rotation at low and high temporal frequencies, but not at medium temporal frequencies.

## Experiment 2: controlling for the stimulus visibility

The first experiment showed age-related sensitivity loss to direction of all fractal rotation temporal frequencies using a stimulus composed of only low spatial frequencies to avoid the known age-related sensitivity loss to high spatial frequencies. Using such a stimulus, we assumed that the age-related effect to fractal rotation was due to motion processing *per se*, not to a lower visibility of the noise. The goal of the second experiment was to confirm this empirically. To differentiate between an age-related sensitivity loss to feature tracking motion processing *per se* from a visibility loss, we measured the sensitivity to a stationary (i.e., not rotating) noise pattern.

### Materials and methods

#### Subjects

The same subjects as in the first experiments participated in the second experiment.

#### Stimuli

**Figure 4:**
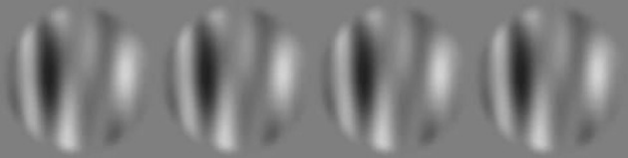
**Example of a vertical stimulus presented in the first-order control condition**. A sequence of four presented frames on a single interval is shown. The same noise image is presented at all frames.

**Figure 5:**
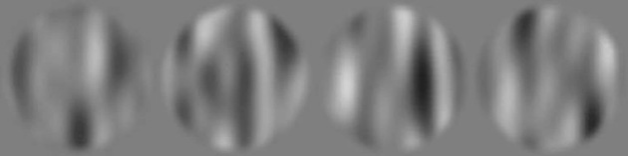
**Example of a vertical stimulus presented in the fractal control condition**. A sequence of four presented frames on a single interval is shown. Noise is resampled on every presented frame. As can be seen, a flickering pattern is obtained.

Replicas of first-order and fractal rotation stimuli used in the first experiment were presented to subjects, except that the orientation did not change over time. Hence, a single noise frame was generated for first-order rotation control condition resulting in a static noise pattern rich in orientation cues. For the fractal rotation control stimulus, noise frames were refreshed at every frame (60 Hz), resulting in dynamic noise rich in orientation cues. For both stimuli, the mean orientation of the spatial filter was randomly assigned a value of 0 or 90°. Importantly, both stimuli used in this control condition are accessible to first-order sensitive mechanisms. Examples of presented stimuli are given in Figures 4, 5.

#### Procedure

A single interval, two-alternative-forced choice procedure was used in combination with an orientation discrimination task (horizontal or vertical). As in the first experiment, contrast thresholds were measured using a 2-down-1-up staircase protocol (Levitt, 1971).

### Results and discussion

For each observer, contrast sensitivity for first-order and fractal rotation controls was obtained from logarithmic transformed contrast sensitivity values. Average contrast sensitivity for discrimination of orientation of first-order and fractal rotation was then calculated. Results show similar log contrast sensitivities between both age groups for first-order (younger adult = 2.17 ± 0.031, older adult = 2.16 ± 0.032) and fractal rotation (younger adult = 2.44 ± 0.030, older adult = 2.39 ± 0.032) control conditions. Independent samples t-tests indicate no significant effect of age on log sensitivity to both static [*t*_(20)_ = 0.063, *p* = 0.950] and dynamic [*t*_(20)_ = 1.078, *p* = 0.294] noise pattern. In other words, the visibility of the static and dynamic noise was similar for both age groups so the age-related sensitivity losses to first-order and fractal rotation observed in the previous experiment must be due to motion processing *per se*, not to some other factor affecting stimulus visibility.

## General discussion

The temporal sensitivity functions to first-order and fractal rotation were band-pass and low-pass in nature, which is consistent with our previous findings (Lagacé-Nadon et al., 2009) that led us to conclude that first-order and fractal rotation stimuli are analyzed by the first-order and feature tracking motion systems, respectively. Because fractal rotation can be designed to solicit the feature tracking motion system in the absence of high spatial frequency content, it constitutes an appropriate stimulus to study high-order motion processing in the elderly (who are less sensitive to high spatial frequencies). Results indicate an age-related sensitivity loss to fractal rotation at all temporal frequencies and an age-related sensitivity loss to first-order motion at low and high temporal frequencies but not at medium temporal frequencies. As confirmed in the second experiment, the sensitivity loss to fractal rotation was not attributable to a lower visibility of the noise pattern, which implies that this age-related sensitivity loss was due to feature tracking motion processing *per se*. To our knowledge, the current study is the first to provide direct evidence of an age-related sensitivity loss to feature tracking motion processing.

The age-related sensitivity loss to feature tracking observed here is in accordance with the results of two other studies (Habak and Faubert, 2000; Tang and Zhou, 2009). As such, Habak and Faubert (2000) found a sensitivity loss at all temporal frequencies for second-order motion processing, but only at low and high temporal frequencies for first-order motion processing. Age had no significant impact on first-order motion sensitivity at a medium temporal frequency (4 Hz). More recently, Tang and Zhou (2009) found that age-related sensitivity loss to second-order motion processing began earlier and was more pronounced than the sensitivity loss to first-order motion processing. As mentioned in the introduction, the existence of a second-order motion system remains controversial. The results from these two studies have shown a particular age-related sensitivity decline to second-order motion processing which could be reflecting a deficit to the feature tracking motion system rather than the second-order motion system as assumed by the authors. The current findings that age affects feature tracking are also compatible with this alternate hypothesis. Consequently, if there is a second-order motion system, the question of whether it is particularly sensitive to aging remains open.

Our results diverge from the ones obtained by Billino et al. (2011) who found similar age-related sensitivity loss to first- and second-order motion processing. However, it is likely that their second-order motion stimulus was processed by the first-order motion system. First, they tested at a relatively high temporal frequency (~7 Hz) and it has been argued that the second-order motion at high temporal frequencies can be processed by the first-order motion system (Holliday and Anderson, 1994; Ukkonen and Derrington, 2000; Allard and Faubert, 2008a, 2013a) Second, they tested in the peripheral visual field and there is some evidence suggesting that there is no motion system other than the first-order motion system under such conditions (Allard and Faubert, 2013b). And more critically, Billino and collaborators have used a motion *detection* task that did not require any second-order motion processing. Subjects had to indicate which of the four simultaneously presented stimuli contained motion. Since adding contrast-defined motion (i.e., a second-order stimulus) to a static texture introduces drift-balanced first-order motion (i.e., the same amount of expected first-order motion drifting in the same and opposite directions as the second-order motion, Chubb and Sperling, 1988), subjects could perform the task simply by detecting the first-order motion. Other studies have generally used a motion *discrimination* task, which requires second-order motion processing since although such a stimulus contains first-order motion, it is drift-balanced and therefore is not informative of the drifting direction of the second-order motion. Consequently, for any of these three reasons it is likely that Billino et al. (2011) second-order motion stimuli were processed by the first-order motion system, which would explain their findings of similar age-related deficits to first- and “second-order” motion processing.

Another way to probe the first-order and feature tracking motion systems is with short- and long-range apparent motion, respectively (Braddick, 1974). As such, Roudaia et al. (2010) have looked at the effect of aging on motion processing using random-dot kinematograms in a two-frame apparent motion paradigm and systematically varied the spatial step-size and the interstimulus interval between the two frames. They found that the elderly were less accurate than their youth at discriminating the motion direction when the spatial or temporal spacing was high (>0.3 degrees or >40 ms). At such high spacing, apparent motion was found to be process by a correspondence-based (i.e., feature tracking) motion system (>0.25 degrees, Braddick, 1974; and >40 ms, Georgeson and Harris, 1990). Thus, although Roudaia et al. (2010) claimed that they were investigating the low-level (first-order) motion system, the age-related effects they found for long-range apparent motion are more likely due to high-level motion processing. This is compatible with the current findings of an age-related sensitivity loss due to the feature tracking motion system.

The current study shows that aging affects high-level, feature tracking motion processing. Previous studies evaluating the effect of aging on “high-level” motion processing other than second-order have generally focused on MOT and global motion processing. As mentioned in the introduction, MOT is sensitive to aging (Trick et al., 2005; Sekuler et al., 2008; Kennedy et al., 2009), but since MOT probes various high-level processes (e.g., divided attention, observer's strategy) it is difficult to determine which of these is specifically affected by aging. Conversely, feature tracking has the advantage of being a high-level, attention-based task without directly soliciting all these factors. Thus, the feature tracking appears to be relatively simple for a high-level attention-based task and the fact it is affected by aging supports the hypothesis of a general age-related decline of attention-based processing.

Global motion processing requires the integration of local (typically first-order) motion over a large area. A recent review by Hutchinson et al. (2012) concludes that aging impairs global motion processing only under some conditions: when the visual field is very large, at low and high (but not medium) velocities and at low contrast. There are at least two reasons that could suggest that age-related sensitivity loss to global motion processing may not reflect a deficit to high-level *global motion* processing *per se*. The first issue relates to the size of the visual field stimulated. As suggested by Faubert (2002) integrating information over larger visual field areas may require the solicitation of larger neural networks and by its very nature could show age-related defects not seen for smaller integration zones. A good example of this is the study by Legault et al. (2012), where they demonstrated that biological motion perception for increasingly larger stimuli became more and more difficult for the elderly while the young observer generally maintained a stable level of performance. There is also evidence from the dual-task paradigms, such as the useful field of view, that visual field size matters in aging (Ball et al., 1988; Sekuler et al., 2000). Hence, the age-related sensitivity loss to global motion, when using a very large stimulus, could depend on integration over a critical field size and not on the global *motion* processing *per se*. Another reason why age-related global motion sensitivity loss may not necessarily reflects an impairment to global motion processing *per se* is that global motion consists in integrating local first-order motion. Thus, lower sensitivity to local first-order motion processing may result in a lower sensitivity to global motion. As mentioned above, age-related global motion sensitivity loss was observed at low and high velocities, but not at medium velocities. In the current study and another by Habak and Faubert (2000), analogous results for local first-order motion processing were observed: aging caused an age-related sensitivity loss at low and high temporal frequencies, but not at medium temporal frequencies. Consequently, it is possible that age-related sensitivity loss to global motion at low and high velocities could be due to a sensitivity loss to local first-order motion processing rather than *global* motion processing *per se*. Note that this could also explain the different age-related effects observed by Billino et al. (2008) for various high-level motion tasks requiring the integration of moving dots (i.e., translational global motion, radial global motion and biological motion). Indeed, these different age-related effects (for instance, aging was found to affect translational but not radial global motion) could be due to the fact that different dot speeds were used for different tasks and that aging affects differently local motion processing at different speeds. Conversely, the current study found an age-related sensitivity loss that must be due to a high-level motion processing (i.e., feature tracking) at all temporal frequencies, including the ones at which first-order motion processing was spared. Thus, compared to global motion processing studies, our study clearly shows an age-related sensitivity loss to *high-level motion* processing *per se*.

Faubert (2002) proposed the complexity hypothesis to explain visual perceptual impairments observed with healthy aging. According to this hypothesis, aging would create subtle diffuse neurobiological alterations. These would have little impact on simple tasks requiring small neural network, but would result in a measurable age effect for complex tasks requiring integration over a broader neural network or for those requiring many processing steps (e.g., inter-attribute spatial frequency discrimination or symmetry perception). The previously observed age-related selective effect on second-order motion processing has been interpreted as evidence in favor of the complexity hypothesis (Habak and Faubert, 2000; Tang and Zhou, 2009). However, the current findings of an age-related impairment to feature tracking and partial sparing of the first-order motion processing does not confirm nor infirm this complexity hypothesis. Nevertheless, there is no doubt that feature tracking is more complex, requires more processing steps and involves larger neural networks than first-order motion processing. Thus, the selective age-related impairment to feature tracking is compatible with the complexity hypothesis. On the other hand, the fact that first-order motion processing and feature tracking involve qualitatively different motion systems implies that the current results are also compatible with the selective deficit hypothesis. For instance, feature tracking involves attentional resources to track features (Cavanagh, 1992). Therefore, the choice of first-order and feature tracking is not optimal for directly addressing the complexity hypothesis for motion *per se*. Given that aging affects attention (Kramer and Kray, 2006; Kramer and Madden, 2008), the effect of aging on feature tracking could relate to a selective attentional deficit.

## Conclusions

The present study confirms previous studies (Habak and Faubert, 2000; Tang and Zhou, 2009) showing selective second-order motion sensitivity losses. However, what was particularly relevant in the present study is that: (1) the motion system (feature tracking) processing this second-order motion stimuli was known and not controversial, and (2) the stimuli were void of high-spatial frequency components known to be affected by aging. We found an age-related deficit on second-order motion processing at all temporal frequencies including the ones for which no age-related effect on first-order motion processing was observed. We conclude that aging affects the ability to track features.

### Conflict of interest statement

The authors declare that the research was conducted in the absence of any commercial or financial relationships that could be construed as a potential conflict of interest.

## References

[B1] Allard R. Faubert J. (2008a). First- and second-order motion mechanisms are distinct at low but common at high temporal frequencies. J. Vis. 8, 1–17 10.1167/8.2.12 18318638

[B2] Allard R. Faubert J. (2008b). The noisy-bit method for digital displays: converting a 256 luminance resolution into a continuous resolution. Behav. Res. Methods 40, 735–743 10.3758/BRM.40.3.735 18697669

[B3] Allard R. Faubert J. (2013a). No second-order motion system sensitive to high temporal frequencies. J. Vis. 13, 1–14 10.1167/13.5.4 23559594

[B4] Allard R. Faubert J. (2013b). No dedicated second-order motion system in the periphery, in Conference Proceedings and Abstracts (Naples, FL).

[B5] Anstis S. M. Mather G. (1985). Effects of luminance and contrast on direction of ambiguous apparent motion. Perception 14, 167–179 10.1068/p140167 4069947

[B6] Ball K. K. Beard B. L. Roenker D. L. Miller R. L. Griggs D. S. (1988). Age and visual-search-expanding the useful field of view. J. Opt. Soc. Am. A 5, 2210–2219 3230491 10.1364/josaa.5.002210

[B7] Bennett P. J. Sekuler A. B. McIntosh A. R. Della-Maggiore V. (2001). The effects of aging on visual memory: evidence for functional reorganization of cortical networks. Acta Psychol. (Amst.) 107, 249–273 10.1016/S0001-6918(01)00037-3 11388138

[B8] Bennett P. J. Sekuler R. Sekuler A. B. (2007). The effects of aging on motion detection and direction identification. Vision Res. 47, 799–809 10.1016/j.visres.2007.01.001 17289106

[B9] Benton C. P. O'Brien J. M. Curran W. (2007). Fractal rotation isolates mechanisms for form-dependent motion in human vision. Biol. Lett. 3, 306–308 10.1098/rsbl.2007.0056 17360252 PMC2464696

[B10] Billino J. Bremmer F. Gegenfurtner K. R. (2008). Differential aging of motion processing mechanisms: evidence against general perceptual decline. [Research Support, Non-U.S. Gov't]. Vision Res. 48, 1254–1261 10.1016/j.visres.2008.02.014 18396307

[B11] Billino J. Braun D. I. Bremmer F. Gegenfurtner K. R. (2011). Challenges to normal neural functioning provide insights into separability of motion processing mechanisms. [Research Support, Non-U.S. Gov't]. Neuropsychologia 49, 3151–3163 10.1016/j.neuropsychologia.2011.07.009 21807009

[B12] Braddick O. J. (1974). A short-range process in apparent motion. Vision Res. 14, 519–527 10.1016/0042-6989(74)90041-8 4423193

[B13] Cavanagh P. (1992). Attention-based motion perception. Science 257, 1563–1565 10.1126/science.1523411 1523411

[B14] Cavanagh P. Mather G. (1989). Motion: the long and short of it. Spat. Vis. 4, 103–129 10.1163/156856889X00077 2487159

[B15] Chubb C. Sperling G. (1988). Drift-balanced random stimuli: a general basis for studying non-Fourier motion perception. J. Opt. Soc. Am. A 5, 1986–2007 10.1364/JOSAA.5.001986 3210090

[B16] Chubb C. Sperling G. (1989). Two motion perception mechanisms revealed through distance-driven reversal of apparent motion. Proc. Natl. Acad. Sci. U.S.A. 86, 2985–2989 10.1073/pnas.86.8.2985 16594030 PMC287045

[B17] Crassini B. Brown B. Bowman K. (1988). Age-related changes in contrast sensitivity in central and peripheral retina. Perception 17, 315–332 10.1068/p170315 3067210

[B18] Crum R. M. Anthony J. C. Bassett S. S. Folstein M. F. (1993). Population-based norms for the Mini-Mental State Examination by age and educational level. JAMA 269, 2386–2391 10.1001/jama.1993.03500180078038 8479064

[B19] Della-Maggiore V. Sekuler A. B. Grady C. L. Bennett P. J. Sekuler R. McIntosh A. R. (2000). Corticolimbic interactions associated with performance on a short-term memory task are modified by age. J. Neurosci. 20, 8410–8416 11069948 10.1523/JNEUROSCI.20-22-08410.2000 PMC6773167

[B20] Elliott D. Whitaker D. MacVeigh D. (1990). Neural contribution to spatiotemporal contrast sensitivity decline in healthy ageing eyes. Vision Res. 30, 541–547 10.1016/0042-6989(90)90066-T 2339508

[B21] Faubert J. (2002). Visual perception and aging. Can. J. Exp. Psychol. 56, 164–176 10.1037/h0087394 12271747

[B22] Georgeson M. A. Harris M. G. (1990). The temporal range of motion sensing and motion perception. [Research Support, Non-U.S. Gov't]. Vision Res. 30, 615–619 10.1016/0042-6989(90)90072-S 2339514

[B23] Habak C. Faubert J. (2000). Larger effect of aging on the perception of higher-order stimuli. Vision Res. 40, 943–950 10.1016/S0042-6989(99)00235-7 10720665

[B24] Holliday I. E. Anderson S. J. (1994). Different processes underlie the detection of second order motion at low and high temporal frequencies. Proc. R. Soc. Lond. B Biol. Sci. 257, 165–173 10.1098/rspb.1994.0111

[B25] Hutchinson C. V. Arena A. Allen H. A. Ledgeway T. (2012). Psychophysical correlates of global motion processing in the aging visual system: a critical review. [Review]. Neurosci. Biobehav. Rev. 36, 1266–1272 10.1016/j.neubiorev.2012.02.009 22343109

[B26] Jackson G. R. Owsley C. McGwin G. Jr. (1999). Aging and dark adaptation. Vision Res. 39, 3975–3982 10.1016/S0042-6989(99)00092-9 10748929

[B27] Kennedy G. J. Tripathy S. P. Barrett B. T. (2009). Early age-related decline in the effective number of trajectories tracked in adult human vision. J. Vis. 9, 21.1–21.10 10.1167/9.2.21 19271931

[B28] Kline D. W. Schieber F. Abusamra L. C. Coyne A. C. (1983). Age, the eye, and the visual channels: contrast sensitivity and response speed. J. Gerontol. 38, 211–216 10.1093/geronj/38.2.211 6827038

[B29] Kramer A. F. Kray J. (2006). Aging and attention, in Lifespan Cognition: Mechanisms of Change, eds Bialystok E. Craik F. I. M. (Oxford; Toronto: Oxford University Press), 57–69 10.1093/acprof:oso/9780195169539.003.0005

[B30] Kramer A. F. Madden D. J. (2008). Attention, in The Handbook of Aging and Cognition, 3rd edn, eds Craik F. I. M. Salthouse T. A. (New York, NY: Psychology Press), 189–249

[B31] Lagacé-Nadon S. Allard R. Faubert J. (2009). Exploring the spatiotemporal properties of fractal rotation perception. [Comparative Study Research Support, Non-U.S. Gov't]. J. Vis. 9:3 10.1167/9.7.3 19761318

[B32] Legault I. Troje N. F. Faubert J. (2012). Healthy older observers cannot use biological-motion point-light information efficiently within 4 m of themselves. Iperception 3, 104–111 10.1068/i0485 23145271 PMC3485817

[B33] Levitt H. (1971). Transformed up-down methods in psychoacoustics. J. Acoust. Soc. Am. 49 Suppl. 2:467+ 10.1121/1.1912375 5541744

[B34] Lu Z.-L. Sperling G. (1995). The functional architecture of human visual motion perception. Vision Res. 35, 2697–2722 10.1016/0042-6989(95)00025-U 7483311

[B35] Lu Z.-L. Sperling G. (2001). Three-systems theory of human visual motion perception: review and update.[see comment][erratum appears in *J. Opt. Soc. Am. A Opt. Image. Sci. Vis.* 2002;19, 413]. J. Opt. Soc. Am. A Opt. Image Sci. Vis. 18, 2331–2370 10.1364/JOSAA.18.002331 11551067

[B36] McIntosh A. R. Sekuler A. B. Penpeci C. Rajah M. N. Grady C. L. Sekuler R. (1999). Recruitment of unique neural systems to support visual memory in normal aging. Curr. Biol. 9, 1275–1278 10.1016/S0960-9822(99)80512-0. 10556091

[B37] Morrison J. D. McGrath C. (1985). Assessment of the optical contributions to the age-related deterioration in vision. Q. J. Exp. Physiol. 70, 249–269 2989968 10.1113/expphysiol.1985.sp002907

[B38] Owsley C. Sekuler R. Siemsen D. (1983). Contrast sensitivity throughout adulthood. Vision Res. 23, 689–699 10.1016/0042-6989(83)90210-9 6613011

[B39] Raghuram A. Lakshminarayanan V. Khanna R. (2005). Psychophysical estimation of speed discrimination. II. Aging effects. J. Opt. Soc. Am. A. Opt. Image Sci. Vis. 22, 2269–2280 10.1364/JOSAA.22.002269 16277296

[B40] Roudaia E. Bennett P. J. Sekuler A. B. Pilz K. S. (2010). Spatiotemporal properties of apparent motion perception and aging. J. Vis. 10, 1–15 10.1167/10.14.5 21131565

[B41] Seiffert A. E. Cavanagh P. (1999). Position-based motion perception for color and texture stimuli: effects of contrast and speed. Vision Res. 39, 4172–4185 10.1016/S0042-6989(99)00129-7 10755155

[B42] Sekuler A. B. Bennett P. J. Mamelak M. (2000). Effects of aging on the useful field of view. Exp. Aging Res. 26, 103–120 10.1080/036107300243588 10755218

[B43] Sekuler R. McLaughlin C. Yotsumoto Y. (2008). Age-related changes in attentional tracking of multiple moving objects. [Research Support, N.I.H., Extramural]. Perception 37, 867–876 10.1068/p5923 18686706

[B44] Smith A. T. (1994). Correspondence-based and energy-based detection of second-order motion in human vision. J. Opt. Soc. Am. A Opt. Image Sci. Vis. 11, 1940–1948 10.1364/JOSAA.11.001940 8071735

[B45] Smith A. T. Ledgeway T. (1997). Separate detection of moving luminance and contrast modulations: fact or artifact? Vision Res. 37, 45–62 10.1016/S0042-6989(96)00147-2 9068830

[B46] Tang Y. Zhou Y. (2009). Age-related decline of contrast sensitivity for second-order stimuli: earlier onset, but slower progression, than for first-order stimuli. [Research Support, Non-U.S. Gov't]. J. Vis. 9, 18 10.1167/9.7.18 19761333

[B47] Trick L. M. Perl T. Sethi N. (2005). Age-related differences in multiple-object tracking. [Comparative Study Research Support, Non-U.S. Gov't]. J. Gerontol. B Psychol. Sci. Soc. Sci. 60, P102–P105 10.1093/geronb/60.2.P102 15746018

[B48] Tulunay-Keesey U. Ver Hoeve J. N. Terkla-McGrane C. (1988). Threshold and suprathreshold spatiotemporal response throughout adulthood. J. Opt. Soc. Am. A 5, 2191–2200 10.1364/JOSAA.5.002191 3230489

[B49] Ukkonen O. I. Derrington A. M. (2000). Motion of contrast-modulated gratings is analysed by different mechanisms at low and at high contrasts. Vision Res. 40, 3359–3371 10.1016/S0042-6989(00)00197-8 11058734

[B50] Weale R. A. (1975). Senile changes in visual acuity. Trans. Ophthalmol. Soc. U.K. 95, 36–38 1064207

[B51] Wilson H. R. Ferrera V. P. Yo C. (1992). A psychophysically motivated model for two-dimensional motion perception. Vis. Neurosci. 9, 79–97 10.1017/S0952523800006386 1633129

